# Multiscale Analyses of Mammal Species Composition – Environment Relationship in the Contiguous USA

**DOI:** 10.1371/journal.pone.0025440

**Published:** 2011-09-27

**Authors:** Rafi Kent, Avi Bar-Massada, Yohay Carmel

**Affiliations:** 1 Faculty of Civil and Environmental Engineering, The Technion – Israel Institute of Technology, Haifa, Israel; 2 Department of Forest and Wildlife Ecology, University of Wisconsin–Madison, Madison, Wisconsin, United States of America; Duke University, United States of America

## Abstract

Relationships between species composition and its environmental determinants are a basic objective of ecology. Such relationships are scale dependent, and predictors of species composition typically include variables such as climate, topographic, historical legacies, land uses, human population levels, and random processes. Our objective was to quantify the effect of environmental determinants on U.S. mammal composition at various spatial scales. We found that climate was the predominant factor affecting species composition, and its relative impact increased in correlation with the increase of the spatial scale. Another factor affecting species composition is land-use–land-cover. Our findings showed that its impact decreased as the spatial scale increased. We provide quantitative indication of highly significant effect of climate and land-use–land-cover variables on mammal composition at multiple scales.

## Introduction

Understanding the factors that affect the distribution of biodiversity in time and space is a central objective of ecology [1]. Relationships between environmental variables (e.g., climate, topography) and biodiversity patterns are scale-dependent, both spatially and temporally [2]. Species richness, probably the most studied aspect of biodiversity, has often been shown to vary as a function of spatial scale [3], [4].

Theories concerning the mechanisms governing distribution patterns of biodiversity measures range from global (latitudinal species richness gradient) to very local scales, and relate to environmental, historical and evolutionary processes [5]. Most studies concentrate on species richness as a measure of biodiversity, resulting in various theories and hypotheses offering mechanistic explanations for patterns of species richness. These explanations are related to interspecific interactions and climate conditions [6], energy levels [7], area effects [8], neutral theory [9] and others. However, the mechanistic explanations for species richness patterns do not necessarily extend to explanations of species composition patterns. Two areas may hold a similar number of species, while the identity of the species might differ considerably, rendering species richness of little value to differentiate between them [10]. Recently, it has been suggested that species richness patterns are largely determined by historical-biogeographical processes [11].

Here we focus on patterns of species composition, rather than species richness. Theories on species composition patterns include a neutral model, which suggests that all variations are caused by random differences in the dispersal of demographically and competitively equal species [9], [12]; an environmental model, which relates species distributions to environmental conditions [13]; and a model that claims that species composition is determined by interspecific interactions within and between trophic levels [14].

Although species composition has rarely been studied at multiple spatial scales, there are exceptions such as the studies of Grand and Cushman [15] and Grand and Mello [16], in which scale was defined qualitatively, i.e., plot, patch and landscape scale. However, most studies on species composition were restricted to a single scale [17], [18], [19], [20]. Applied across multiple scales, multivariate analyses may provide a wider picture of the relationships between environmental variables and species composition [21], [22]. Understanding species composition – environment relationships, and specifically how they are affected by spatial scale, may improve the ability of conservationists to predict both the spatial distribution of biodiversity, and its reaction to global and regional changes [23].

There are serious conceptual and practical impediments to such analyses. A central conceptual challenge is the nature of scale [24]. Scale is characterized by both grain (grid cell size) and extent [25]. In most studies, a change of only a single element of scale is regarded as a full change of scale [26]. Here we used a “complete” approach, in which both grain and extent are modified together in the process of upscaling (see Appendix S1 for details).

The major practical impediment for such analyses is data availability [27]. Presence-absence data are only available for relatively small extents [27]. Presence-only (occurrence) data in large quantities and for diverse taxonomic groups have become available in the last decade via data portals such as the Global Biodiversity Information Facility (GBIF) and other portals that allow easy access to digitized databases, mostly based on museum and university collections [28]. However, presence-only data are often considered improper for such analyses, due to a range of inherent biases [29], [30]. The validity of using presence-only data in ecological analyses has been studied repeatedly in the context of modeling the distribution of a single species or modeling species richness patterns, but results are inconclusive [31]. In a previous study [32], we evaluated the reliability of using presence-only data for studying multiscale diversity patterns based on taxonomic or functional group composition. The assessment confirmed that presence-only data are sufficient for analyzing the relationships between species composition and environmental determinants. The objective of the current study was to quantify the variation in the relationships between mammal species composition and its environmental determinants, at varying spatial scales. More specifically, we hypothesized that climate is the predominant environmental factor affecting species composition at large scales. An additional hypothesis was that land-use and land-cover (LULC) variables are highly influential at fine spatial scales, however, when grain size is large enough to contain all (or most) of the possible LULC types, the effect of those variables will diminish. Regarding topography and primary productivity, we hypothesized that their effect will be more prominent at small scales.

## Results

### Variable group effects

**Figure 1:**
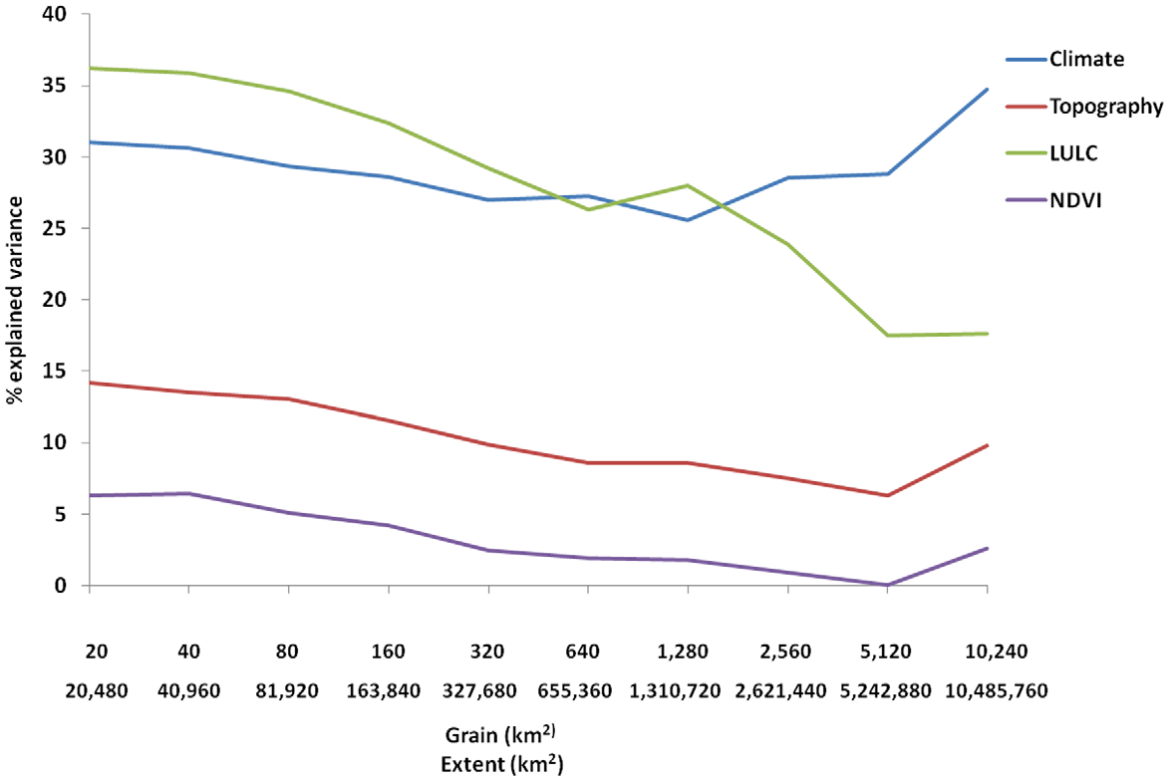
Explained variance rates of environmental variable groups in mammal species composition at varying spatial scales in the contiguous USA (demonstrated using CCA analyses). Scale consists of grain size (upper number on the x-axis) and extent (lower number on the x-axis). Explained variances represent the pure effect of each variable group used in the analyses (see Table 1 for details on the different variable groups).

**Figure 2:**
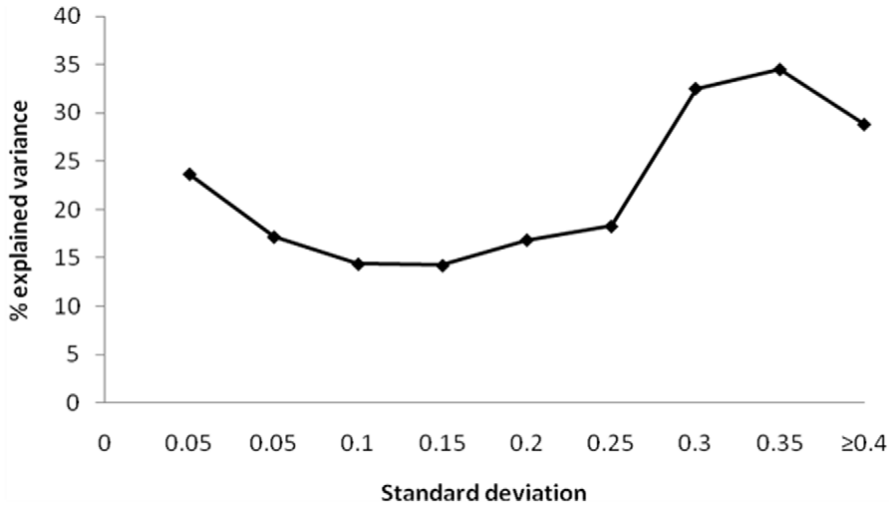
Mean values of standard deviation in environmental variables (for detailed description of variables see Table 1). For convenience, values are presented as averages in intervals of 0.05. Pearson's correlation between SD and % explained variance is 0.44.

Climate and Land use – Land cover (LULC) variables explained the largest amount of variance in community composition at all spatial scales of the analyses (Fig. 1). The amount of variance explained by LULC variables decreased gradually until the seventh scale (grain size 1,280 km^2^,,extent 1.3*10^6^ ), and then dropped sharply from ∼30% of the total explained variance to ∼15% between scales 7 and 9. Climate, which explained a slightly smaller proportion of the variance in species composition than LULC variables at the six smaller six scales, also showed a decrease in the amount of explained variance until scale 7, but then exhibited an increase between scales 7 and 10. Topography and primary production explained a relatively small amount of variance in species composition at all the analyzed scales. In general, the proportion of explained variance in species composition decreased with increasing scale, except at the largest scale, where both variable groups exhibited a moderate increase (Fig. 1). The correlation between effective gradient length of the different variable groups and the proportion of variance in species composition explained by each group was intermediate (Pearson's r = 0.44, Fig. 2).

### Within - group analyses

**Figure 3:**
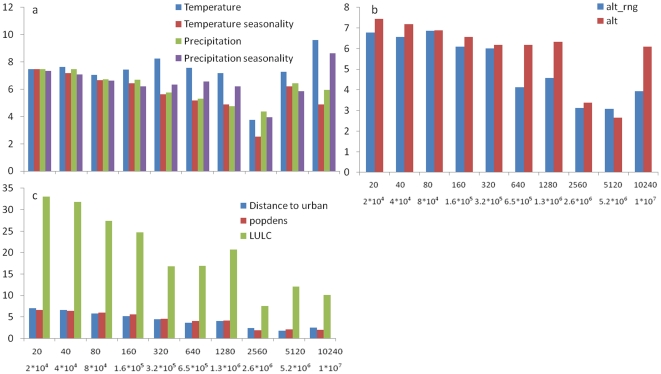
Explained variance rates of individual environmental variables in mammal species composition: a) climatic variables; b) topographic variables and c) LULC variables. Land cover is the combined effect of six land cover categories (agriculture, forestry, open herbaceous, urban, water and wetland). Prec_sea and Temp_sea stand for *precipitation seasonality* and *temperature seasonality* respectively; Altitude –rng stands for altitude range; DTU stands for *distance to nearest urban area*; and Pop density stands for *population density*.

Within the group of climatic variables, all four variables had equal contribution to the variance explained by the group at the four smallest scales (Fig. 3a). However, at the larger scales mean annual temperature was the predominant climatic feature. Precipitation seasonality also explained a relatively high proportion of the total explained variance at three of the five largest scales (Fig. 3a).

**Figure 4:**
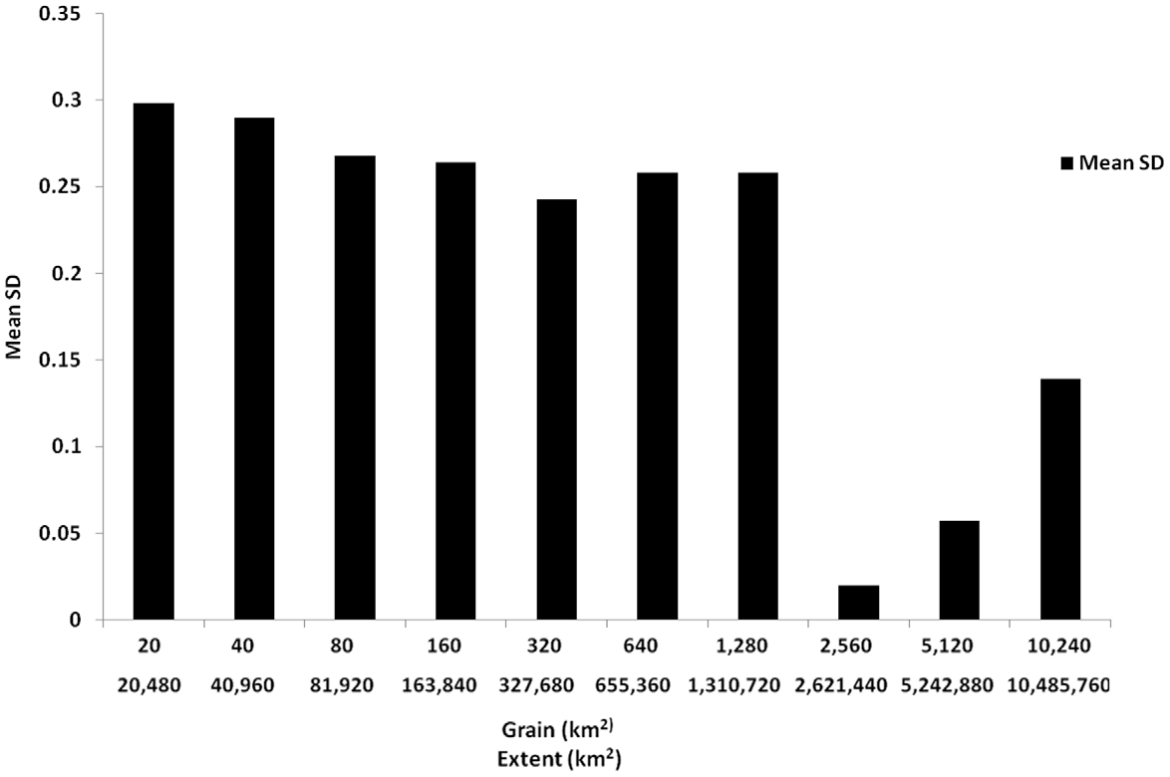
Mean value of standard deviation in land-cover variables (for detailed description of variables see **Table 1**) per spatial scale analyzed.

In the topography group, Altitude generally explained a larger proportion of the variance in species composition compared to altitude range. However differences were relatively small at the small scales and larger at the larger scales (Fig. 3b). Land-cover variables in the LULC group explained most of the variance in that group at all scales (Fig. 3c). However, when we plotted the mean standard deviation in land cover variables, within the LULC groups (i.e., agriculture, forestry etc.), we found that the decrease in the amount of variance explained by LULC variables (Fig. 1) corresponded to a sharp decrease in the variance in the land cover variables at the eighth scale (Fig. 4).

## Discussion

Our analyses revealed that at grain sizes of 10^1^ to 10^5^ km^2^ and extents from 10^5^ to 10^8^ km^2^ respectively, mammal species composition is affected largely by climate and LULC variables. LULC variables had sizeable influence on species composition at the smaller scales, probably via habitat degradation and fragmentation, and ultimately, habitat loss [33]. Topography was not a prominent factor in these analyses, but it is probably more important at finer scales [34]. These results partially corroborate our hypotheses. As we hypothesized, climate is indeed a predominant factor affecting mammal species composition within the contiguous USA. However, at smaller scales, LULC variables are more influential, and explain a larger amount of variance in species composition than climate. This is consistent with theoretical predictions that at fine scales, effects of climatic determinants are obscured by biological interactions and that the effect of climate becomes more evident at larger scales [26]. Also corroborating our hypotheses is our finding that, at the largest scales, as the variance within sampling units increases and the variance between them decreases, LULC become less explanatory. We found intermediate correlation levels between the effective gradient lengths (for an explanation of effective gradient length please see Methods and Materials section) and the amount of variance in species composition explained by the different variable groups. This suggests that there is a change in the reaction of species composition to environmental gradients varies among scales, although some of that change is attributable to differences in gradient lengths. Wiens [26] described a phenomenon called scale-domains, based on a review of studies that used different sized quadrats to study patterns of plant distributions. He suggested that the change in distribution patterns of ecological phenomena observed with scale is monotonous within each scale-domain. In contrast, between domains, pattern variability is chaotic and unpredictable, manifested as high variability between sampling units. Accordingly, our findings indicate that between the seventh and eighth scales there is a possible a shift from one scale domain to another, in both climate and LULC variable groups (Fig. 1). The existence of scale domains is possibly indicated here by the shift in the direction and slope of the line in Figure 2. The high levels of explained variance attributed to land-cover (i.e., forestry, agriculture, urban area, etc.) suggest that at all scales, land cover type is the predominant human related factor affecting mammal species composition.

This study, to the best of our knowledge, is among the first to analyze the relationships between species composition and the environmental conditions that affect it at large and multiple spatial scales [but see for example 35]. We found that scale was a prominent factor in these relationships, having a greater impact than that of geographical factors that affect environmental conditions within each scale. This line of research has the potential to contribute much to the understanding of global biodiversity patterns. Studying other taxonomic groups and other regions of the world would be an important step towards establishing a knowledge base of these relationships, which in turn may serve to test general biogeography theories.

## Materials and Methods

Our data consisted of all occurrence records found in the GBIF portal [36] of terrestrial mammals (excluding bats) in the contiguous USA. All data were downloaded from GBIF during March–June 2009. Bats were excluded from the analyses under the assumption that the ecological demands and responses of bats to environmental variables may be very different than all other mammals, and thus may decrease the probability of elucidating coherent answers to our questions. Our dataset consisted of ∼308,000 records, including 284 species. It originated from ∼70 datasets within GBIF. We used all geo-referenced records of specimens and observations in the datasets, with the exception of records with less than four decimal digits in at least one coordinate (either latitude or longitude).

In addition to mammal occurrence data, we compiled environmental data related to 15 variables, which we categorized according to 4 groups: climate; topography; land-use/land-cover (LULC); and primary productivity (Table 1). The spatial resolution of all environmental layers was (or was reduced to) 0.0833° (∼10 km). As a measure of anthropogenic disturbance we measured the average distance to nearest urban area in 0.00833° (∼1 km) grid in the entire study area, using the Euclidean distance function in ArcMap [37] with a polygonal urban area layer (see Table 1). We then calculated the mean value of the distance to nearest urban area in each cell in the grid, at each spatial scale. Seasonality in climatic variables, i.e. temperature and precipitation (Table 1), was represented by inter-month variance. The data were downloaded as GIS layers from Worldclim [38]. The coefficient of variation (CV) was the measure of variance used to represent precipitation seasonality. Seasonality in temperature was represented as standard deviation, as CV makes no sense when values are between −1 and 1. For more details see the Worldclim website http://www.worldclim.org/bioclim.

**Table 1:** Environmental variables used in the analyses, and their source.

Variable name	Description	Source
Temperature		Worldclim [38]
Temperature seasonality	Standard deviation of monthly temperature values	
Precipitation		
Precipitation seasonality	Coefficient of variation of monthly precipitation values	
Altitude		Worldclim [38]
Alt_Range		
NDVI		MODIS – http://glcf.uniacs.umd.edu/data/ndvi
Pop-Density	Population density	FAOGeoNetwork http://www.fao.org/geonetwork/em/mainhome
Urban*	Urban area	
Forestry*	Forest	
Open-Herbaceous*	Herbaceous vegetation	
Agriculture*	Agricultural area	
Water*	Large water body	
Wettland*	Wetland area	
Distance to Urban	Distance to nearest urban area calculated at a fine resolution (0.0083°) and averaged for each grid-cell	Data were extracted from ESRI data files

*denotes values based on % coverage of categorical variables.

To analyze the effects of scale on community composition, we wrote an ArcGIS python script that generated sets of rectangular sampling units of extent *E* and grain *g* at each scale (Table 2). At each scale, the area of a grid cell is twice the area of a cell in the previous scale. The value of each environmental variable in a cell was calculated as the average of the respective values in all pixels contained within that cell. In each sampling unit, the script counted the number of pixels that had species observations in them and the total number of species in those observations. We then identified sampling units that had sufficient information for a Canonical Correspondence Analysis (the CCA) analysis by setting thresholds for numbers of species and pixels with observations. For the subsequent statistical analysis, we only used sampling units that had more than five species and at least 30 pixels with non-singleton observations. For each sampling unit that complied with the thresholds, and at each scale, we ran a partial Canonical Correspondence Analysis (pCCA) using the vegan package [39] in the R statistical software package, version 2.12 [40]. The difference between CCA and pCCA is that pCCA decomposes the explained variance to its components, i.e. it allows determining how much of the variance is explained by individual variables or variable groups. This is accomplished by using the variable(s) of interest as constraints (i.e. explanatory variables) and the rest of the environmental variables as conditioning variables (also termed co-variables). Thus, the proportions of the variance explained by the conditional variables alone and by the interactions between the variable(s) of interest and the conditioning variables, are accounted for [for a detailed description of pCCA see 21]. We ran pCCA for each variable using the vegan package (Oksanen et al. 2011) in the R statistical software package, version 2.12 (R Development Core Team 2010). For pCCA, we split the environmental variables into four groups: climate (mean annual temperature, temperature seasonality, mean annual precipitation and precipitation seasonality), topography (elevation and elevation range); land-use land-cover (distance to urban areas, population density, and percentages of agriculture, forest, grasslands, urban, surface waters, and wetland areas); and NDVI. We then ran pCCA for each group separately, using its variables as the constraints, while using all other variables as conditioning variables [13], [21], [22], [41]. In addition we analyzed each individual variable as the constraint, using all other variables as conditioning variables, in order to differentiate the various variables within each group. To calculate the amount of variance in species composition explained by each variable and each group, we divided the inertia of each group in each sampling unit by the overall inertia in the respective sampling unit, and multiplied it by 100. Total inertia is an expression of the amount of variance in the species data within the sampling units [22], and individual inertia is equivalent to the amount of variance that is related solely to the specific variable or group of variables, after accounting for the variance explained by other variables and the interaction between the different variables [21].

**Table 2:** A list of sampling units used in the analyses, including grain size (side length in km) and extent (area in km^2^).

Scale	grain (km)	extent (km^2^)	valid samples (#)
1	4.47	20,480	9
2	6.32	40,960	12
3	8.94	81,920	12
4	12.65	163,840	14
5	17.89	327,680	13
6	25.30	655,360	9
7	35.78	1,310,720	7
8	50.60	2,621,440	7
9	71.55	5,242,880	3
10	101.19	10,485,760	2

In order to discriminate between the effect of effective environmental gradient length and the amount of variance in species composition that is explained by that gradient, we calculated effective gradient lengths of the different variable groups within the sampling units. Effective gradient length is related to the amount of variance of a variable within the entire dataset. We calculated the effective gradient length by standardizing all variables so that they ranged between 0 and 1, and calculating the cumulative standard deviation within each variable group, which is the standard deviation in each variable group across all scales. Next we correlated that group's variance with the amount of variance that was explained by that group. High correlation coefficient values indicate strong effect of effective gradient length while low correlation values suggest effect of scale independent of differences in gradient length. In addition, in order to understand the sharp decrease in the amount of species composition variance explained by land-cover variables within the LULC variable group, we calculated the mean value, over the five different categories of land-cover (agriculture, forestry, open-herbaceous, urban and water), and then over the different units, of the standard deviation in those variables. A decrease in that value would indicate that the level of variance within the units is decreasing, i.e. each unit is composed of more components. A sharp decrease in intra-unit variance of land-cover variables should manifest as a decrease in the explanatory power of that group, in explaining the variance in species composition.

## Supporting Information

Appendix S1 **An explanation of the concept of simultaneous alteration of grain and extent when using multiple spatial scales in ecological studies.**
(DOCX)
