# Supplementary material for: Multiscale Analyses of Mammal Species Composition – Environment Relationship in the Contiguous USA
Source: PLoS One. 2011 Sep 27;6(9):e25440. doi: 10.1371/journal.pone.0025440 (PMC3181324; doi:10.1371/journal.pone.0025440)
Supplement: Appendix S1 — An explanation of the concept of simultaneous alteration of grain and extent when using multiple spatial scales in ecological studies. (DOCX) [file pone.0025440.s001.docx]

**Appendix S1**

The issue of spatial scale in ecological studies has been the subject much debate [e.g. 24,26,42,43]. It has become unequivocal that scale plays an important role in the effect environmental conditions have on ecological states and processes, and thus on patterns of biodiversity distribution. Due to the complex nature of scale, it is not trivial to evaluate its impact, disentangling it from other factors. In particular, scale consists of two major elements, grain and extent, and a change of scale may be a change in grain, or in the extent, or both. This complexity was rarely treated explicitly in previous efforts to study the impact of scale [43].

Here we used a systematic upscaling approach, in which we kept a constant ratio between grain and extent, in order to alter both the lengths of environmental gradients, and the basic ecological unit of the analysis. This approach holds a principal advantage over exclusively altering either one of the components of scale. Some environmental variables are heterogeneous at small scales (i.e. change over short geographical distances, such as soil type for example) while others vary over large geographical distances (e.g. climatic variables). While altering a single component of scale may detect patterns of either small or large scale variables, it will probably fail in detecting the opposite. Moreover, some variables may change over intermediate distances, and some may be heterogeneous at multiple scales. Thus, only a systematic alteration of both components of scale may detect the effect of variables at all levels of heterogeneity. Furthermore, by using an unbalanced rescaling scheme, one is expected to receive partially erroneous results, leading to inaccurate conclusions, as patterns are expected to deviate from their true form (Kent and Carmel, unpublished data).
